# Inception and movement of a ‘subgrain boundary precursor’ in ice under an applied stress, observed by X-ray synchrotron radiation Bragg imaging^1^

**DOI:** 10.1107/S1600576715006342

**Published:** 2015-04-25

**Authors:** A. Philip, L. Capolo, J. Meyssonnier, J. Baruchel

**Affiliations:** a Université Grenoble Alpes, LGGE, CNRS, BP 96, Grenoble 38041, France; b European Synchrotron Radiation Facility, BP 220, Grenoble 38043, France

**Keywords:** ice, single-crystal Bragg diffraction, dislocations, deformation mechanism

## Abstract

Basal slip of dislocations, the easiest deformation mechanism of ice crystals, does not allow a response to any strain state. The first steps of another mechanism, with a moving subgrain boundary precursor region, which permits accommodating the effect of an applied load, is investigated on an ice single crystal, mainly using synchrotron radiation Bragg diffraction imaging. During this process, the evolution of the local integrated intensity shows that there is both a general multiplication of dislocations within the crystal and a movement of basal dislocations towards the surface. The ‘subgrain boundary precursor’ region evolves towards a classical grain boundary when further deformed.

## Abstract 2

Both optical microscopy with polarized light and polychromatic beam synchrotron X-ray diffraction imaging (white-beam topography) are used to study *in situ* the way an ice single-crystal deforms.

## 1. Introduction   

**Figure 1:**
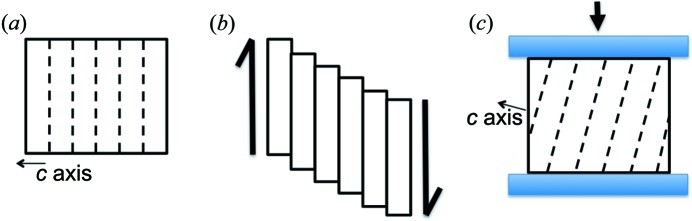
Schematic diagrams of (*a*) a single crystal of ice, (*b*) its directional deformation leading to glides in the basal plane and (*c*) the boundary conditions imposed by a rigid-blade displacement, which are not compatible with a homogeneous distribution of the basal glides.

The viscoplastic behaviour of crystals is associated with the presence, movement and creation of dislocations, which allow crystals to deform under applied stresses. The movement of dislocations occurs in a very directional way in anisotropic crystals. In the case of ice, a highly anisotropic crystal, this movement is very easy within the basal (0001) plane of the hexagonal structure and very difficult in directions perpendicular to this plane (Petrenko & Whitworth, 1999 ▶). This is illustrated schematically in Fig. 1 ▶(*a*), showing that, under a shear stress, directional deformation easily occurs by gliding along basal slip planes, where dislocations move or form.

The study of the deformation of ice is interesting not only because it constitutes the basic mechanism of the movement of glaciers and polar ice sheets, but also because ice is a model material for deformation studies of a wide range of anisotropic materials crystallizing in a non-cubic structure, for instance trigonal or hexagonal crystals (Duval *et al.*, 2010 ▶; Barsoum *et al.*, 1999 ▶; Gharghouri *et al.*, 1999 ▶).

An anisotropic single crystal submitted to kinematic boundary conditions (namely, displacement boundary conditions; Fig. 1 ▶) not compatible with the basal glide will develop heterogeneities. This occurs when the principal directions of the applied stress do not coincide with the symmetry axes of the anisotropy (off-axes tests). In this case, it is known that non-homogeneous deformation develops because the constraints imposed by the experimental device do not allow enough degrees of freedom (Boehler *et al.*, 1987 ▶, 1994 ▶).

To accommodate any homogeneous deformation of a crystalline material, five independent slip systems are needed (Taylor, 1938 ▶). In ice, since the basal plane provides only two independent systems, other mechanisms are then needed to provide additional degrees of freedom. Homogeneous deformation can only occur in rather unusual ‘academic’ cases. One example is constituted by a monocrystalline sample (ellipsoidal inclusion) completely surrounded by an isotropic matrix. This situation was reproduced experimentally by Mansuy *et al.* (2001 ▶) when studying a large cylindrical ice single crystal embedded in a fine-grained randomly oriented ice matrix that can be considered as macroscopically isotropic. This experimental configuration corresponds to the inclusion case of Eshelby (1957 ▶), because it removes the kinematic boundary conditions far away from the single crystal and so avoids strain localization caused by applying the load directly on its boundaries. Thus, uniform distributions of the activated basal glides develop in the main single crystal, so the deformation can be considered as macroscopically homogeneous.

Within a polycrystal, to ensure strain compatibility between grains with different lattice orientations, heterogeneities develop, such as kink and bending bands (Wilson & Zhang, 1996 ▶; Mansuy *et al.*, 2000 ▶; Lebensohn *et al.*, 2009 ▶). A recent X-ray imaging study of the inception of the deformation of a high-quality crystalline ice grain belonging to a triple-crystal sample provides a good example of these heterogeneities: it shows that slip planes develop during the very first steps of the deformation, and that later on a very deformed region, a precursor of a subgrain boundary, develops in the neighbourhood of the triple junction (Philip *et al.*, 2013 ▶). This subgrain boundary-like region arises because of the strain incompatibilities associated with the very directional basal glides of the crystalline grains.

In the present work, we are concerned with a realistic but still simple case: a single crystal loaded by compression under the plane strain condition, or more exactly by a pressure applied through a rigid blade constrained to glide in an imposed direction, between two rigid plates enclosing the sample. The displacement of the non-deformable blade along a fixed direction induces strain incompatibilities at the blade–crystal boundary, which are reminiscent, in a simpler way, of those associated with the contact with another crystalline grain.

The aim of the present paper is thus to investigate, in a single crystal, the inception of a process similar to that observed in the three-crystal sample, *i.e.* the creation and evolution under load of a moving subgrain/grain boundary. In order to carry out this investigation we used two complementary probes, polarized light X-ray microscopy and poly­chromatic Bragg diffraction imaging (X-ray topography).

## 2. Samples and experimental techniques   

**Figure 2:**
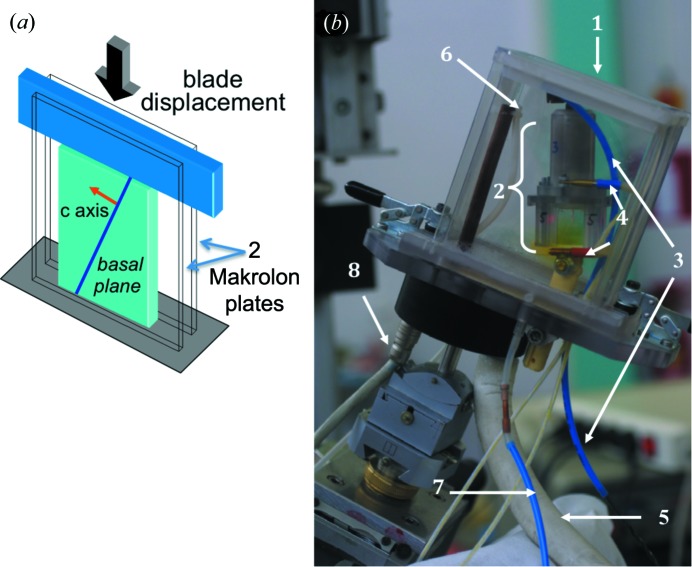
(*a*) A schematic diagram of the setup that allows the loading of a crystal under the plane strain condition, used for both the optical and X-ray imaging observations. (*b*) The experimental device, with (1) the refrigerated cell, (2) the setup for the mechanical test, (3) the compressed-air apparatus for loading, (4) the temperature sensors, (5) the tube for the supply of ‘cold’ nitrogen, (6) the nitrogen release, (7) the return of ‘hot’ nitrogen and (8) the power cable.

Parallelepiped-shaped (21 × 17 mm largest surface and 1 mm thick) ice crystals were milled in such a way that the *c* axis lay in the largest surface plane of the specimen (±3°). The crystals were cut within large grains of a polycrystalline columnar S2 type (Michel & Ramseier, 1971 ▶) obtained in a unidirectional temperature gradient by freezing deionized and demineralized water on a cold table (Baruchel *et al.*, 2013 ▶). Creep experiments on these platelet-shaped sections were carried out under the plane strain condition with a specially designed device (Fig. 2 ▶). The load is applied through a blade of the same thickness as the sample, sliding between two Makrolon plates. This device, which is transparent to both visible light and X-rays (absorption and diffraction), prevents sublimation. The Makrolon plates are in contact with the largest surfaces of the platelet, ensuring non-deformation in the direction perpendicular to these plates (plane strain state), the two small lateral surfaces being free to move (Fig. 2 ▶ *a*). The ice thickness of 1 mm was chosen to limit the superposition of features on the image of the white X-ray diffracted beam, recorded on film.

Our samples initially had their *c* axis at 75° of the applied load direction, since this favours basal-plane activation. In addition, this configuration facilitates the experimental implementation for *in situ* X-ray diffraction topography (diffractometer tower position, cold temperature and compression devices). A nitrogen flux is used to maintain the temperature of the sample environment within the range 263 ± 3 K.

Both optical microscopy with polarized light and polychromatic beam synchrotron radiation Bragg diffraction imaging (white-beam topography) were used to study *in situ* the way the crystal distorts. Optical microscopy allows following the evolution of the shape of the deformed crystal and its local birefringence. White-beam topography was employed to measure, with high angular and spatial sensitivity, the mis­orientations between regions of the sample and the evolution of crystalline defects during the deformation process.

**Figure 3:**
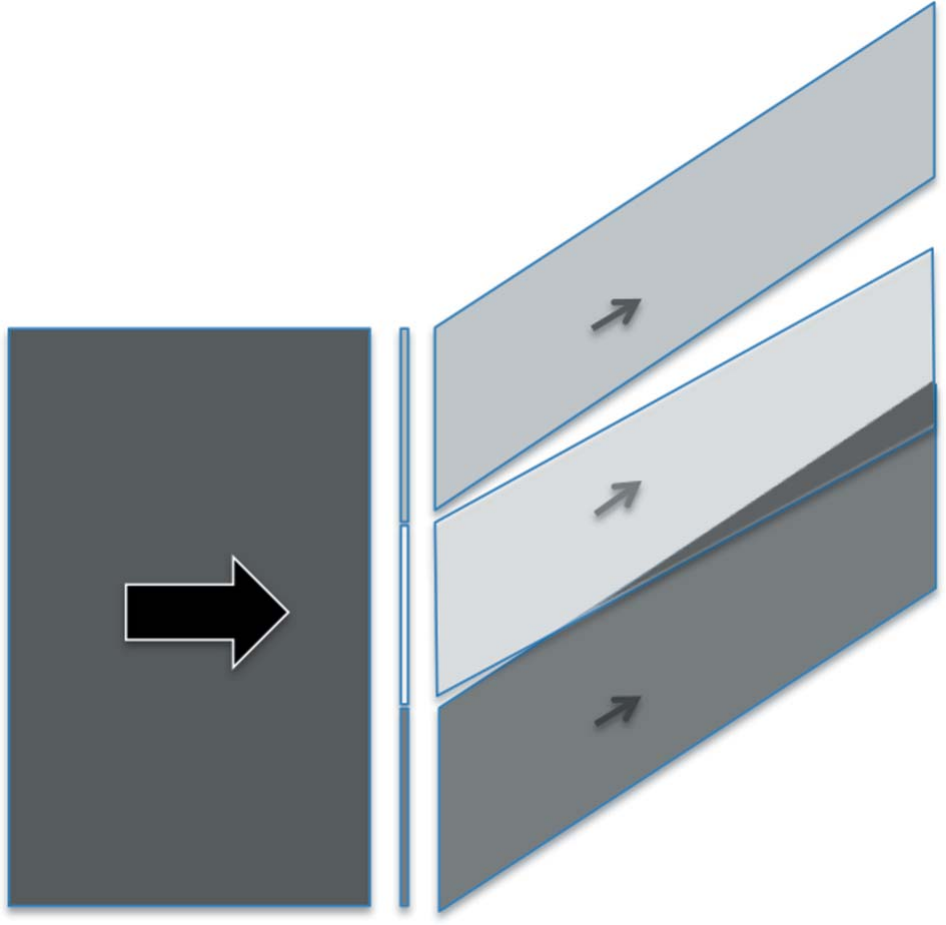
A schematic drawing showing, in white-beam imaging, the incident polychromatic beam and, for a given Bragg spot, the diffracted beams associated with three subgrains that compose the sample. The images can be superimposed or separated at the detector level.

The use of a polychromatic beam is justified by the fact that all illuminated regions of the sample, whatever their orientation, will be exposed to X-ray photons with an energy suited to fulfilling the Bragg law, and the distortion of the resulting image with respect to the shape of the illuminated crystal area provides information on the relative misorientations of the various regions of the sample. In particular, subgrain boundaries produce images that can be superimposed or separated at the detector level, as indicated schematically in Fig. 3 ▶. On the other hand, inspecting within a given Bragg spot allows the observation of contrast, which is associated with defects within the sample. In order to preserve the spatial resolution of the image, the polychromatic beam should be nearly parallel at the level of the sample [see, for instance, Authier (2001 ▶), and references therein]. Polychromatic nearly parallel beams of sufficient energy are available at modern synchrotron facilities, and in particular at the ESRF, where the experiments were carried out. In addition, we note that our ice sample only exhibits a very faint absorption of the X-ray beam (μ_t_ ≃ 0.12) for the diffracted energies (∼20 keV) corresponding to the observed Bragg spots.

**Figure 4:**
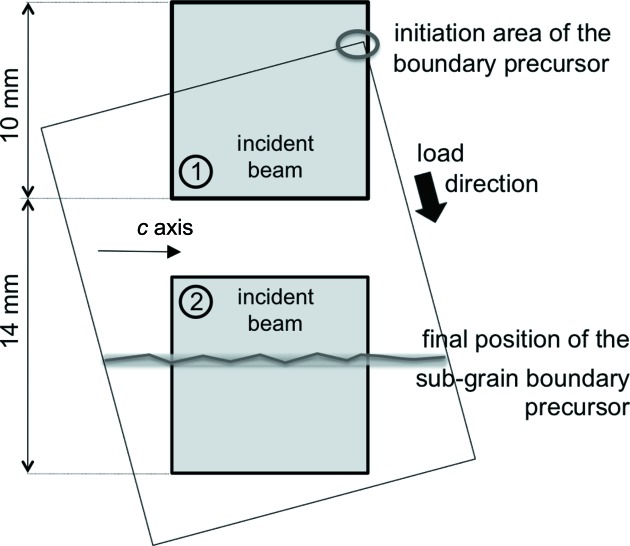
A schematic diagram showing the two areas of the single-crystal sample that were observed at various steps of the loading process. The subgrain boundary precursor was initiated in a corner of the sample, and then during loading it glides downward.

Several crystals were analysed, but we restrict this paper to the results observed on two samples with roughly the same *c*-axis direction with respect to the load, the first being studied by polarized light and the second by diffraction topography. Two areas of the single-crystal sample were studied more particularly and they are indicated in Fig. 4 ▶. The results obtained appear to be representative of those obtained on all the samples with the same crystallographic orientation.

## 3. Experimental results   

### 3.1. Optical observations   

**Figure 5:**
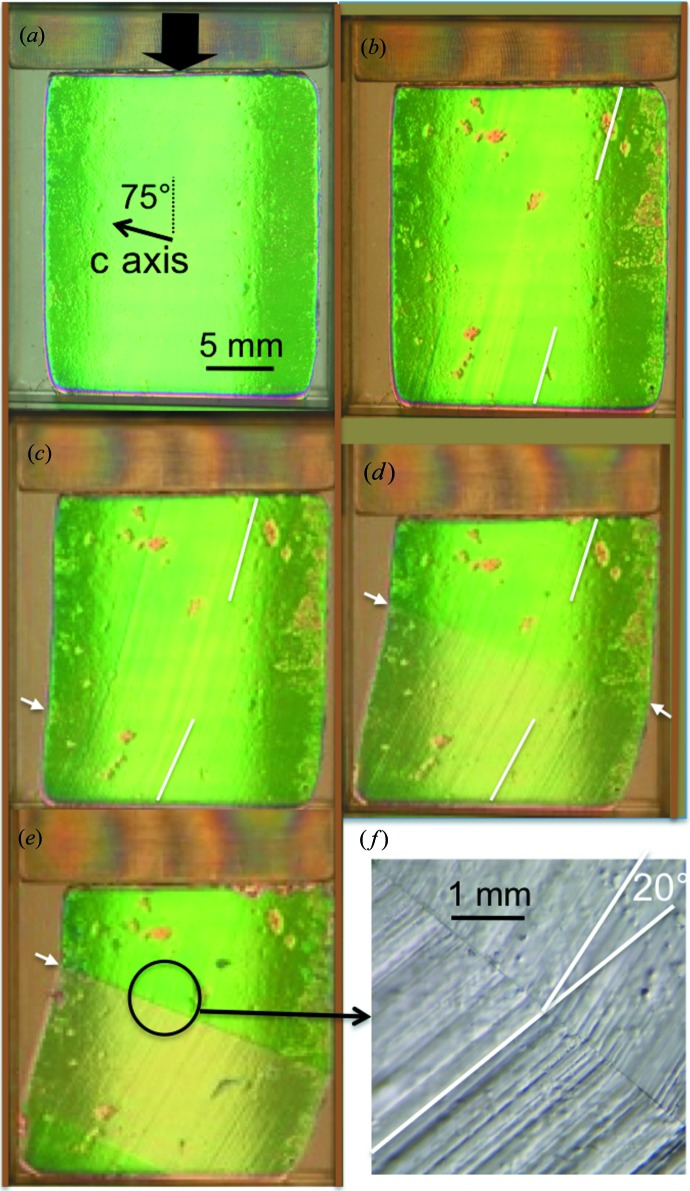
Evolution of the optical image with polarized light of an ice single crystal confined within a refrigerated cell and submitted to an external load imposed by the moving upper blade. (*a*) The initial state, *t* = 0, then σ = 0.25 MPa at (*b*) *t* = 0 h 40 min, (*c*) *t* = 1 h 30 min, (*d*) *t* = 4 h 45 min and (*e*) *t* = 13 h 30 min. (*f*) Magnification of the grain-boundary area.

Fig. 5 ▶ summarizes a series of polarized-light optical microscopy observations on ice crystals under an applied load. Fig. 5 ▶(*a*) corresponds to the initial non-deformed state. The *c* axis and the applied load directions are indicated in this figure. The first features associated with the deformation apparent on the optical micrographs are, as expected, slip planes parallel to the basal plane (Fig. 5 ▶ *b*). This first step is in keeping with the viscoplastic anisotropy of ice. However, the deformation induced by the testing device is not expected to lead to a homogeneous strain field in the crystal, and the crystal accommodates the blade movement through additional mechanisms. When increasing the load, the shape of the crystal is modified to depart from the original rectangle, exhibiting non-straight borders in the direction of loading (Figs. 5 ▶ *c*, 5 ▶ *d* and 5 ▶ *e*). The angle between the upper and lower parts of these borders, noticeable in Fig. 5 ▶(*c*) and easily observable in Figs. 5 ▶(*d*) and 5 ▶(*e*), as well as the associated variation in local birefringence, indicate the occurrence of a grain boundary. This angle increases when increasing the load. Fig. 5 ▶(*f*), the magnified image of Fig. 5 ▶(*e*), shows that the misorientation between the grains, measured through the angle formed by the slip basal planes belonging to the two obtained grains, is about 20°. The left-hand border exhibits a lower angle, because it reached the limit of the cell confining the crystal: this new constraint led to the occurrence of a new grain boundary, visible in the lower left-hand corner of Fig. 5 ▶(*e*).

### 3.2. Synchrotron radiation diffraction imaging observations   

The optical observations indicate the occurrence of two misoriented regions as a response to the applied external load. A very convenient way of studying the inception of this process, quantifying the misorientations between these regions and characterizing the transition region, is, as pointed out above, the *in situ* observation, using a polychromatic synchrotron X-ray beam, of the evolution of the Bragg diffracted images of the ice single crystal at several steps of the application of the external force.

**Figure 6:**
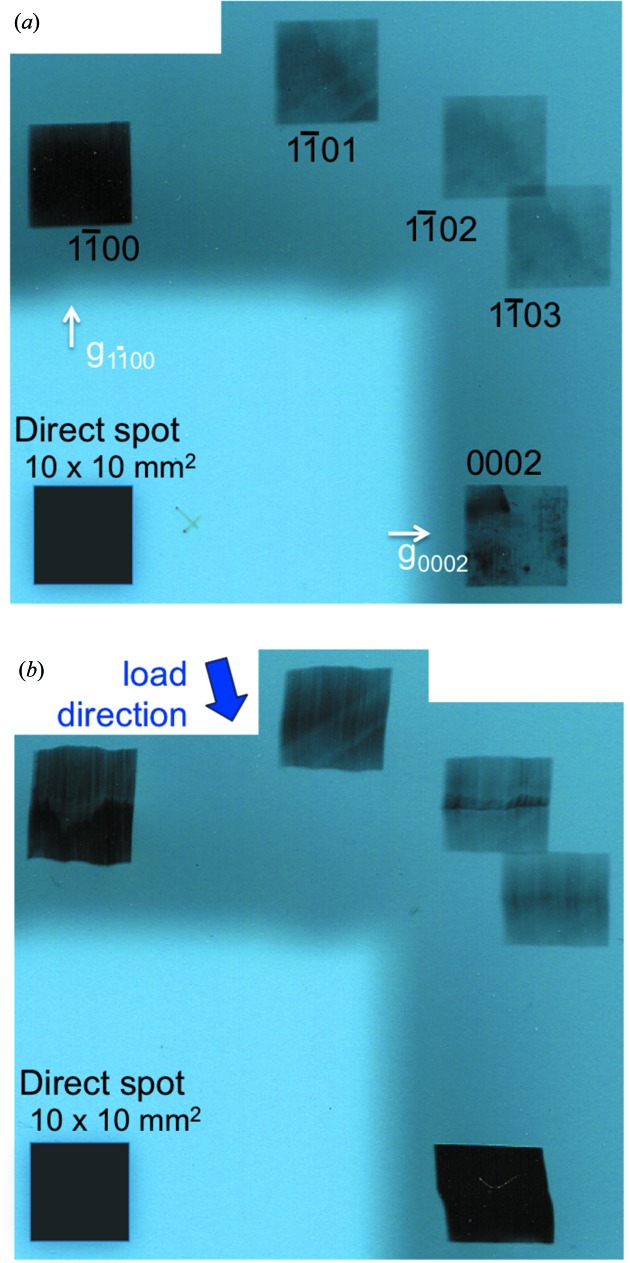
Extended Laue diagrams corresponding to (*a*) the initial state and (*b*) the final state, deformed after 7 h by the application, through the rigid blade, of a series of increasing external loads. The projection of the diffraction vector lies on the direction going from the direct spot to the corresponding diffracted spot; two of these vector projections are explicitly indicated in the figure.

While the study of the evolution of the subgrain boundary location and misorientation requires following its images over several steps of the deformation process, we will first start with a simple comparison of our initial and final states, which already provides a substantial amount of relevant information. To perform this comparison, we consider (Fig. 6 ▶) the images present within the extended Laue diagrams that we obtain when illuminating a large area (10 × 10 mm) of the crystal for both states. Let us first just observe the external shape of the Bragg spots. The detector (in our case an X-ray film) is set parallel to the main surface of the platelet-shaped sample, and the X-ray beam can be considered, within a very good approximation, as parallel. Under these conditions, and taking into account that (i) the thickness *t* of the sample is small with respect to its lateral dimensions and (ii) the Bragg angle θ_B_ is small, in such a way that the projected thickness on the film *t* tanθ_B_ can be neglected with respect to the other dimensions of the image, the shape of the Bragg diffracted image of a non-distorted crystal is equal to the shape of the illuminated area of the sample. This is what is observed in Fig. 6 ▶(*a*): the shapes of the Bragg spot images (the spots have been indexed on this figure) have the same size (10 mm) and shape (square) as the region of the sample illuminated by the X-ray beam. This indicates that, while the crystal in its initial state is not perfect (slip-plane images are already visible in this initial state), its distortion gradient is small enough not to affect the shape of the image.

This is not the case for the Bragg spot images of Fig. 6 ▶(*b*): the 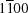 reflection is larger by 15% than the illuminated region in the vertical direction, the 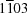 reflection is smaller by about the same amount, and the 0002 spot, corresponding to the basal plane, exhibits a horizontal shift (roughly localized in the middle of the spot) between the upper and lower parts of the image. These features correspond to two misoriented regions (subgrains) in the sample, such that the images are either superimposed or separated, along both the vertical and horizontal directions, as indicated schematically in Fig. 3 ▶.

**Figure 7:**
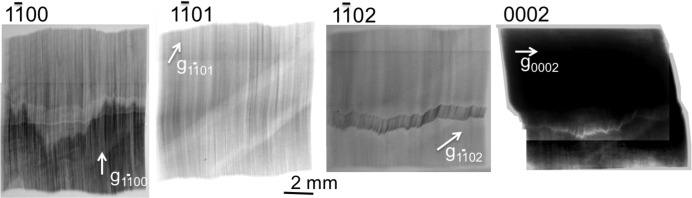
The variation in contrast of the grain sub-boundary for the 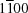, 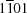, 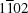 and 0002 reflections after 7 h of loading. The oblique band (particularly visible on the 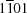 image) corresponds to the absorption of the incident beam by the compressed-air pipe used for the loading.

This figure also indicates that quantitative information can be extracted from simple geometric considerations, knowing that the sample-to-detector distance *D* was ∼32 cm for this experiment. We can therefore infer from Fig. 6 ▶(*b*) that the basal (0002) planes of the two subgrains display an angular shift of ∼5 min of arc, whereas the misorientation of the same subgrains measured on the prismatic planes (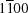) is again ∼5 min of arc. This means that the subgrain boundary we are interested in is associated with both a flexion and a torsion. On the other hand, the contrast of the subgrain boundary is very different in the images of the various collected Bragg spots, as shown in Fig. 7 ▶. In the next section, we will show that, through simulations performed using a program based on a simple geometric mechanism, the main features observed can be explained.

The new subgrain area is initially created in the upper part of the crystal, *i.e.* near the surface in contact with the load-application device (rigid blade), as shown in Fig. 4 ▶. Under increasing applied force, the subgrain grows upward and the sub-boundary moves downward, with a displacement not far from parallel to the basal slip line, up to the point (as the optical observations have already suggested) where it reaches roughly the middle of the sample, where it slows down.

**Figure 8:**
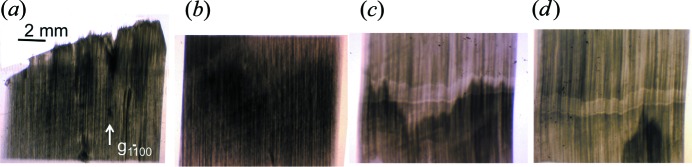
The variation in contrast for the 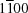 reflection associated with the movement of the subgrain boundary. Just after loading (*a*) in area 1 and (*b*) in area 2 (see Fig. 4 ▶). (*c*) The same area 2, showing the subgrain boundary after 7 h of loading and (*d*) just after unloading.

Fig. 8 ▶ shows a remarkable feature that occurs in all the regions ‘swept’ by the subgrain boundary we are concerned with: we observe in the 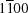 reflection a clear decrease in the integrated intensity in these regions (which manifests through a lightening of these areas on the image, darker signifying more illuminated in our convention), and even in regions of the crystal where the subgrain boundary has not yet arrived. This implies that this crystal has reduced its gradient of distortion in these regions, which could be associated, as will be discussed in the next section, with a reduction in the number of basal dislocations.

**Figure 9:**
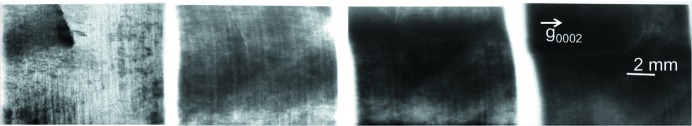
At several steps of loading, the variation in contrast for the 0002 reflection associated with the movement of the subgrain boundary: (*a*) the initial state before loading, (*b*) after 4 h of loading at different pressure applied to the blade piston, (*c*) the same but after 5 h and (*d*) after 6 h of loading.

Fig. 9 ▶ shows that this reduction in integrated intensity under loading is not general at all. On the contrary, when following the basal 0002 reflection we observe a very noticeable increase in integrated intensity over the illuminated area. This means that there is a stronger distortion gradient, namely a greater dislocation density, with a Burgers vector having a component along the *c* axis.

## 4. Discussion   

From its initial state, our sample displays a series of slip-plane images, indicating a high density of basal dislocations (Petrenko & Whitworth, 1999 ▶), which can be estimated to be about 10^6^ cm cm^−3^ (Capolo, 2007 ▶). Under low absorption conditions, and for such a non-perfect crystal, we can neglect, for the further deformed states, contrast mechanisms associated with small departures from the dynamic theory of diffraction, and mainly consider an ‘orientation’ contrast mechanism (Fig. 3 ▶) based only on the superposition or separation of the diffracted beams originating from the volumes of the two subgrains.

**Figure 10:**
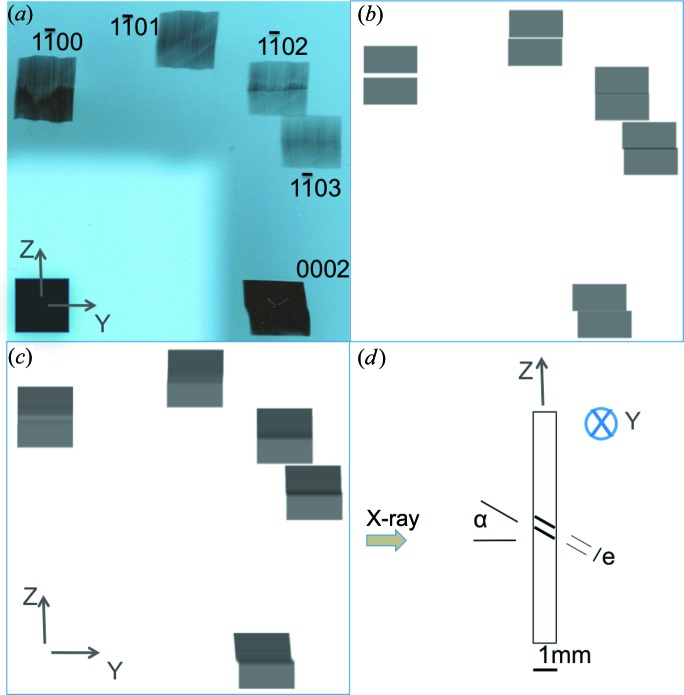
Extended Laue diagrams: (*a*) experimental result after 7 h of loading, and (*b*) and (*c*) simulated patterns assuming two subgrains separated by a boundary with α  =  30° (see text), such that the subgrain boundary is (*b*) a geometric plane, *e*  =  0, or (*c*) a small volume with *e* = 0.5 mm where the misorientation varies continuously from one subgrain orientation to the other. (*d*) A schematic drawing showing the orientation of the subgrain-boundary-like region.

Fig. 10 ▶(*a*) recalls the experimental results corresponding to the final state of deformation, presented in Fig. 6 ▶(*b*). Figs. 10 ▶(*b*) and 10 ▶(*c*) show the results of simulations performed by assuming that the crystal is composed of two subgrains occupying the higher and lower regions of the illuminated sample, separated by a planar boundary displaying an inclination α = 30° with respect to the normal to the largest surface (Fig. 10 ▶ *d*). The lower region is rotated with respect to the higher one by −5 min of arc around the *y* axis and by −5 min of arc around the *z* axis (see Fig. 10 ▶). The diffraction is simply considered as proportional to the illuminated volume in each diffraction spot. These very simple assumptions (geometric rotation between the upper and lower parts of the crystal, and ‘kinematic theory’ type diffraction) produce, as shown in Fig. 10 ▶(*b*), simulated images that describe the main features we can observe in Figs. 6(*b*) and 7.

Nevertheless, some discrepancies remain. The first, clearly visible in Fig. 7 ▶, is that the subgrain boundary is composed not of a single plane but of a series of segments that are only perpendicular on average to the loading direction. This was not included in the simulation software, in order to keep it as simple as possible. A second discrepancy is that the two subgrains, which are, for the simulation of Fig. 10 ▶(*b*), considered as having a uniform orientation, actually display an ‘internal’ small rotation (< 1 min of arc) when going from the upper illuminated region to the subgrain boundary, or from the subgrain boundary to the lower illuminated region. This is indicated by the noticeable inclination of the borders of the basal plane spot, which lie nearly perpendicular to the subgrain boundary. On the other hand, when comparing, again, the basal spots in Figs. 10 ▶(*a*) and 10 ▶(*b*), we see that there is a sharp discontinuity in the simulated image at the level of the subgrain boundary (Fig. 10 ▶ *b*), whereas this image discontinuity is smoothed in the experimental result (Fig. 10 ▶ *a*). These features can be simulated in a very simple way by assuming (i) that the subgrain boundary is not a geometric plane but a volume where the misorientation changes in a sinusoidal way and (ii) that each subgrain displays a rotation of the diffracting planes when going from the upper part of the image to the subgrain boundary region, or, for the other subgrain, from the subgrain boundary to the lower part of the image. Fig. 10 ▶(*c*) shows the simulation we obtain when introducing these additional constraints, which is in very good agreement with the experimental results.

This is also in keeping with the results of previous experiments performed when deforming, in a very similar way, a tricrystalline sample (Philip *et al.*, 2013 ▶). These results clearly showed a continuous small rotation of the diffraction planes and, in the neighbourhood of the triple junction, a region exhibiting an extension of a few hundred micrometres and where the rotation of the planes is one order of magnitude greater than in the remainder of the grain. This distorted region acts as a precursor of a subgrain boundary. The optical experiments of the present work (Fig. 5 ▶) suggest that this region actually evolves towards a more classical subgrain or, when further deformed, a grain boundary.

Fig. 8 ▶ shows that the occurrence and movement of the subgrain boundary is associated with a decrease in the integrated intensity (recorded on the detector when taking a white-beam diffraction image) in the 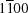 image area already swept by this boundary or in its neighbourhood. This extinction effect [see, for instance, Sabine (1988 ▶)] corresponds, in the low-absorption case we are concerned with, to a reduction in the gradient of distortion in the area where it is produced. The discontinuous ragged shape of the boundary between the high- and low-intensity areas suggests that this is not associated with a long-range strain (such as bending) but, very probably, with a variation in the dislocation density. This reduction can indeed be achieved by a decrease in the number of dislocations (Oleknovich *et al.*, 1983 ▶; Kaganer & Sabelfeld, 2010 ▶). These dislocations should be such that they contribute a distortion of the crystallographic planes used to produce the images, *i.e.* they should have a Burgers vector **b** such that **h·b** is not zero, and consequently the effective distortion of the (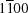) reflecting planes is also not zero (Authier, 2001 ▶). The dislocations with a Burgers vector 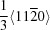 belong to this ensemble. These dislocations glide when the loading is applied and can escape through the largest surface of the sample.

The decrease in the density of dislocations may be explained by the shearing of the crystal parallel to its basal plane induced by the torsion of the sample. A detailed solution for the torsion of a beam with a rectangular cross section made of an anisotropic medium is not straightforward (*e.g.* Whitney & Kurtz, 1993 ▶). However, we can estimate an order of magnitude by considering the torsion of a homogeneous isotropic elastic cylinder around its revolution axis. In this case, with *x* denoting the cylinder axis, the stress tensor has only four nonzero components, *i.e. s*_*xy*_ = *s*_*yx*_ = −*Gaz* and *s*_*xz*_ = *s*_*zx*_ = *Gay*, where *G* is the shear modulus and *a* is the rotation of the cylinder cross section per unit length along **x**. Considering a plane with its normal in the *xz* plane inclined at 45° from the *x* axis, which is approximately the situation of the basal plane in our experiment, this plane is submitted to a shear stress of *s*_*xy*_ cos(π/4) in the **y** direction (which in our experiment corresponds to the direction normal to the largest face of the sample). Adopting approximate values of *G* = 5000 MPa and *a* = 1.4 × 10^−4^ rad mm^−1^ (corresponding to a total torsion of the basal plane of 5 min of arc over 10 mm), the shear stress in the **y** direction varies between 0 and 5 MPa (at *z* = 10 mm). This order of magnitude for the basal shear stress is high enough to induce the movement of basal dis­locations towards the free surfaces of the crystal.

Of course, this reduction in the gradient of distortion under loading is not general. On the contrary, other crystallographic planes exhibit a strong increase in the integrated diffracted intensity, corresponding to the occurrence of a stronger distortion gradient due to the ‘geometrically necessary dis­locations’ (Ashby, 1970 ▶). This is what is observed in Fig. 9 ▶, where a very noticeable increase in integrated intensity is observed in the basal reflection 0002 image. This indicates the occurrence of dislocations with a non-basal Burgers vector, in agreement with the inclination of the borders of the basal plane spot.

Numerical simulations are vital to be truly quantitative regarding these moving subgrain/grain boundaries in ice single crystals, which result from a complex and non-homogeneous stress field imposed by the loading device. Because of this, the resolved shear stress on the different slip systems cannot be determined simply by calculating the Schmid factors for a compression test. These mechanical simulations must take into account the imposed boundary conditions and the anisotropic crystalline character of the ice rheology (Lebensohn *et al.*, 2009 ▶; Mansuy *et al.*, 2002 ▶; Philip *et al.*, 1991 ▶; Capolo, 2007 ▶). The necessary numerical simulations are beyond the scope of the present paper, which aims to show, qualitatively, the physical mechanism associated with the observed deformation in anisotropic ice.

## 5. Conclusions   

In this paper, observations by polarized-light microscopy and white-beam X-ray topography have been discussed for an ice single crystal deformed by the displacement of a non-deformable blade along an imposed fixed direction. Observations and the simulated Laue diagram suggest that a small misoriented volume moves to ensure one more degree of freedom, which is not allowed by a dislocation glide only on the basal plane. The small initial misorientation observed by X-rays, which becomes more and more important during loading, up to its conclusion as a grain boundary, allows the heterogeneous stress field of an off-axes test to be accommodated. We observed similar sub-boundaries nearly perpendicular to the basal plane in other experiments with single crystals differently oriented with respect to loading, and also in grains of loaded multicrystals. This indicates that the displacement of a subgrain-boundary-like region is a mechanism of strain accommodation in ice crystals.
